# Occurrence of *Penicillium brocae* and *Penicillium citreonigrum*, which Produce a Mutagenic Metabolite and a Mycotoxin Citreoviridin, Respectively, in Selected Commercially Available Rice Grains in Thailand

**DOI:** 10.3390/toxins9060194

**Published:** 2017-06-15

**Authors:** Nozomi Shiratori, Naoki Kobayashi, Phitsanu Tulayakul, Yoshitsugu Sugiura, Masahiko Takino, Osamu Endo, Yoshiko Sugita-Konishi

**Affiliations:** 1 Graduate School of Life and Environmental Sciences, Azabu University, 1-17-71, Fuchinobe Chuo-ku, Sagamihara 252-5201, Japan; c521316@gmail.com (N.S.); n-kobayashi@azabu-u.ac.jp (N.K.); sugiura@azabu-u.ac.jp (Y.S.); 2 Faculty of Life and Environmental Sciences, Azabu University, 1-17-71, Fuchinobe Chuo-ku, Sagamihara 252-5201, Japan; endou@azabu-u.ac.jp; 3 Department of Veterinary Public Health, Faculty of Veterinary Medicine, Kasetsart University, Kamphaeng Saen campus, Nakhon Pathom 73140, Thailand; fvetpnt@ku.ac.th; 4 Agilent Technologies, Japan, Ltd., 9-1 Takakura-cho, Hachioji, Tokyo 192-8510, Japan; masahiko_takino@agilent.com

**Keywords:** rice, *Penicillium brocae*, Ames test, citreoviridin, LC-QTOF analysis

## Abstract

Commercially available rice grains in Thailand were examined to isolate the monoverticillate *Penicillium* species responsible for toxic yellowed rice. *Penicillium* species were obtained from seven out of 10 rice samples tested. Among them, one *Penicillium citreonigrum* isolate and six *Penicillium brocae* isolates were morphologically identified. The *P. citreonigrum* isolate produced the mycotoxin citreoviridin on a yeast extract sucrose broth medium. Mycotoxin surveys showed that citreoviridin was not detected in any samples, but one out of 10 rice samples tested was positive for aflatoxin B_1_ at a level of 5.9 μg/kg. An Ames test revealed that methanol extracts from rice grains inoculated with selected *P. brocae* isolates were positive for strains TA100 and YG7108 of *Salmonella typhimurium*, suggesting the presence of base-pair substitution and DNA alkylation mutagens. Our data obtained here demonstrated that aflatoxin B_1_ and toxic *P. citreonigrum* were present on domestic rice grains in Thailand, although limited samples were tested. *Penicillium brocae*, which may produce mutagenic metabolites, was isolated for the first time from the surface of Thai rice grains.

## 1. Introduction

Fungal contamination in food and foodstuffs causes a number of problems in commercially available products, such as food spoilage and food poisoning. In terms of fungi, mycotoxin contamination is of great concern to human health around the world. In Thailand, the major mycotoxins associated with cancer risk found in market commodities are aflatoxin (AF) B_1_ and other aflatoxins [1]. AFB_1_ is a strong class I carcinogen according to the International Agency for Research on Cancer, and several studies have reported rice contaminated with AFB_1_ and other aflatoxins among Thai commodities [2,3,4]. In the postharvest condition, harvested rice grains may be damaged in several ways, such as fungal invasion, mycotoxin contamination, physicochemical changes, and insect activity. Particularly, fungal invasion of stored agricultural products leads to a loss of quality in the storage environment [5]. However, there is currently insufficient information on stored rice as an agricultural commodity in Thailand.

A staple cereal plant, rice (*Oryza sativa* L.) is widely cultivated in Asia, North and South America, the European Union, and Middle Eastern and African countries. Rice provides 20% of the world’s dietary energy supply, while wheat and maize supply 19% and 5%, respectively [6]. The nutritive contents of rice vary depending on the type of rice, but rice is known to be a good source of thiamine, riboflavin and niacin, along with some kinds of amino acids. Thus, the contamination of rice grains with mycotoxins is a serious problem for human health [7]. In Asian countries, the contamination of rice with *Aspergillus* and *Fusarium* toxins such as aflatoxins, trichothecenes, and fumonisins, has been of great concern [8,9], but little attention has been paid to *Penicillium* toxin due to its low presence in rice.

Before and after World War II, the toxic rice problems in Japan occurred after rice was imported, and three historical cases have been documented [10,11]. It was called “yellow rice” and was caused by contamination of rice grains with toxigenic *Penicillium* species. The first case was the isolation of *Penicillium citreonigrum* (formerly, *P. citreo-viride*). Fungus-damaged rice was imported from Taiwan in 1937, and found to be contaminated with the toxic metabolite citreoviridin (CTV), which has been suggested to be responsible for beriberi disease. The second case was an occurrence of *Penicillium islandicum* (current taxon, *Talaromyces islandicus*) in a batch of not yellowed but yellowish-brown rice. This rice was imported from Egypt in 1948. Mycotoxin studies have revealed that *P. islandicum* produces hepatotoxic mycotoxins, such as luteoskyrin, and cyclochlorotine. The third case was caused by the citrinin-producer *Penicillium citrinum*. The rice was imported from Thailand in 1951 [12]. Citrinin is nephrotoxic, but has low acute toxicity. These toxicological characteristics, including the induction of cancer and chemical structures, have been reported by Japanese mycotoxin researchers [10,12].

Recently, an outbreak of beriberi disease occurred in the state of Maranhao, Brazil, and 1207 cases were reported, including 40 deaths. In rice samples from the outbreak region, the neurotoxic mycotoxin CTV was detected at ranges from 0.9 to 31.1 μg/kg. CTV is a known toxic metabolite of *P. citreonigrum* that adversely affects the cardiovascular system, and may be involved in acute cardiac beriberi [13]. However, the relationship between CTV and the disease is still unclear.

In the present study, we examined rice commodities in Thai markets for surveillance of mycoflora. In addition to the alcohol treatment, the rice grains were tested without any treatment in order to assess the stored rice environment in Thailand. Particularly, the presence of the toxic yellowed rice fungus *P. citreonigrum* and related species were surveyed. These monoverticillate species are characterized by slow growth and yellow pigment production on the surface or reverse of the colonies on Czapek yeast agar (CYA) medium. The isolates obtained were tested for CTV production. As an indicator of human toxicity, their metabolites were assayed for mutagenic activities using an Ames test. Contamination of Thai rice samples with mycotoxins was also analyzed.

## 2. Results

### 2.1. Mycoflora on Rice Grains

**Figure 1:**
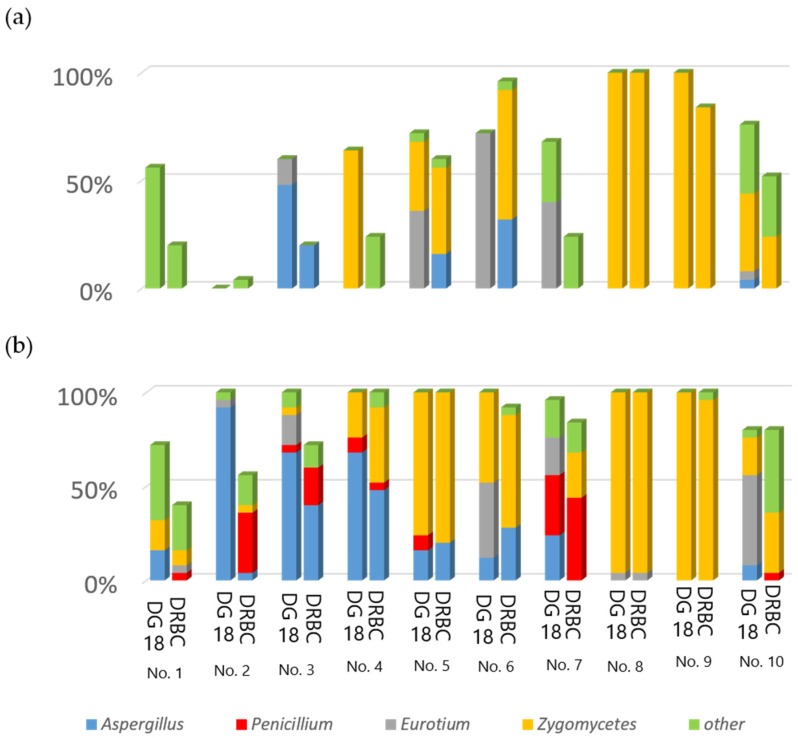
Percentage of fungal genera isolated from 10 samples of Thai rice, which were treated with 70% ethanol exposure (**a**) and without surface disinfection (**b**), and cultured on DG18 (**left**) and DRBC (**right**) media.

Based on macroscopic observation, the developed fungi were classified simply as genera of fungi. In the alcohol-treated group, the developed fungi were *Aspergillus*, *Eurotium* (teleomorph of xerophilic *Aspergillus*), *Mucor* and *Rhizopus* shown as Zygomycetes in Figure 1, and other fungi. Similar findings were noted in the untreated group, except for *Penicillium* also being observed. As shown in Figure 1, the frequency of the genera differed depending on the rice type and region of Thailand. However, almost the same major fungal genera developed between the groups, except for the genus *Penicillium*. Isolates of *Penicillium* species were only found on untreated rice grains. In addition, in the sample from the Surin region, monoverticillate and yellow pigment-producing *Penicillium* isolates were observed. These isolates were subjected to identification at the species level.

### 2.2. Morphological and Phylogenetic Identification

Monoverticillate and yellow pigment-producing *Penicillium* isolates were cultured on CYA and malt extract agar (MEA) media at 25 °C for seven days. Based on the morphological characteristics of *Penicillium* isolates, one isolate was identified as *P. citreonigrum*, but the taxonomic positions of two similar *Penicillium* isolates could not be determined by morphological observation alone. Therefore, these isolates were subjected to a molecular phylogeny analysis.

**Figure 2:**
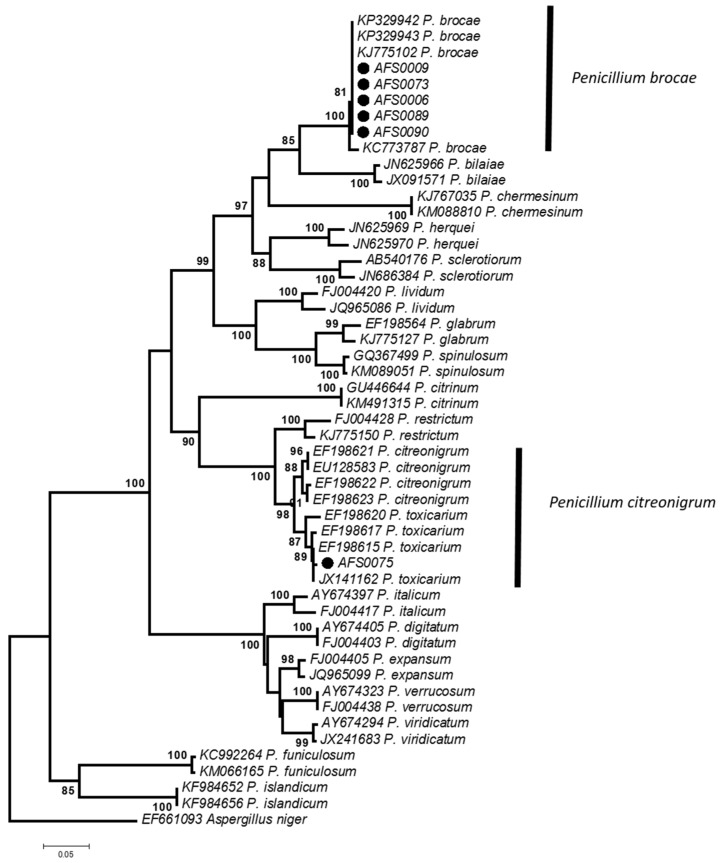
Phylogenetic tree based on the β-tubulin gene. The tree was generated by the neighbor joining method using 1000 bootstrap replicates. Only the bootstrap values above 70% are indicated near the branches. The tree was rooted with the strain of *Aspergillus niger*. The strain numbers marked with black circles are the species obtained in this study. Others were downloaded from GenBank.

Figure 2 shows the NJ-tree inferred from the β-tubulin gene (*β-tub*) of selected *Penicillium* species. Each reference species formed a monophyletic clade with high bootstrap support. The *P. citreonigrum* isolate identified morphologically belonged to *Penicillium toxicarium*, which is included in the clade of *P. citreonigrum*. Recently, *P. toxicarium* was moved to *Penicillium citreosulfuratum* by molecular phylogeny alone [14], and the isolate was suggested to identify as *P. citreosulfuratum*. However, due to finely roughened conidia, especially with roughness arranged in undulation, the isolate differed from the IMI 92228 strain, which is an ex-type of *P. citreosulfuratum* [14,15]. Thus, the isolate was identified as *P. citreonigrum* based on the criterion of *Penicillium* taxonomy by Pitt [15]. Two unknown *Penicillium* isolates were identified as *Penicillium brocae* based on a phylogenetic analysis (Figure 2). The morphological characteristics of these isolates were then compared with the description of *P. brocae* reported by Peterson et al. [16].

**Figure 3:**
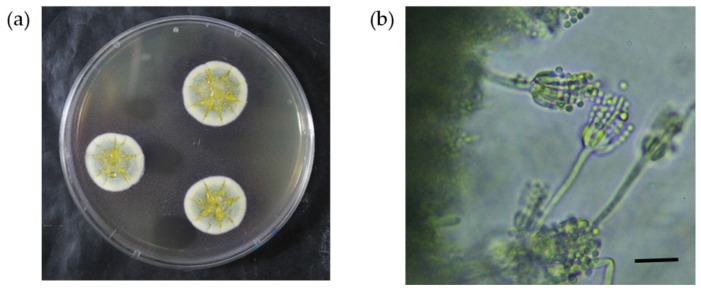
*Penicillium brocae*. (**a**) Colonies grown on CYA medium at 25 °C for 7 days. (**b**) Conidiophores and conidia in a chain. Bar, 10 μm.

As shown in Figure 3, *P. brocae* isolates grew slowly when cultured on CYA at 25 °C for seven days. Colonies were 23–25 mm in diameter, grayish blue-green, velvety, and centrally produced moderate to abundant yellow exudate. The reverse color was yellowish brown or brownish purple, producing soluble naphthalene yellow pigment at the edge. The isolates did not grow at 37 °C. Conidiophores were short and smooth-walled, and vesiculate, monoverticillate penicillus was observed. Phialides were ampulliform, 8–9 × 2–3 μm. Conidia were spherical, 2.5–3.5 µm in diameter, with slightly roughened walls, forming short chains. These characteristics of the observed *P. brocae* isolates were almost the same as the original description of the species.

The sequence data of *P. citreonigrum* AFS-0075, and *P. brocae* AFS-0006 and AFS-0073 strains were deposited in the DNA Data Bank of Japan (DDBJ) under the accession numbers LC216411, LC216406, and LC216408, respectively. 

As relatively few *Penicillium* species were observed, an additional trial was conducted in the same sample but using different rice grains. The trial showed that no *P. citreonigrum* isolate and four *P. brocae* isolates were obtained. Among them, three *P. brocae* isolates were subjected to the molecular phylogeny analysis. The identification of the isolates was confirmed using the *β-tub* region (Figure 2). The sequence data of *P. brocae* AFS-0009, AFS-0089, and AFS-0090 strains were deposited in the DDBJ under the accession numbers LC216407, LC216409, and LC216410, respectively. The strains obtained were not used for further experiments.

### 2.3. Mutagenicity of P. brocae Metabolites

The AFS-0006 and AFS-0073 strains of *P. brocae* were cultured on 20 g of rice grains at 25 °C for 14 days. Early in the culture, the rice was stained yellow, and then covered with abundant green conidia. The cultures were extracted with 100 mL of methanol by sonication for 20 min. The methanol extracts were evaporated to dryness under a nitrogen gas flow and resolved with 5 mL of dimethylsulfoxide.

**Figure 4:**
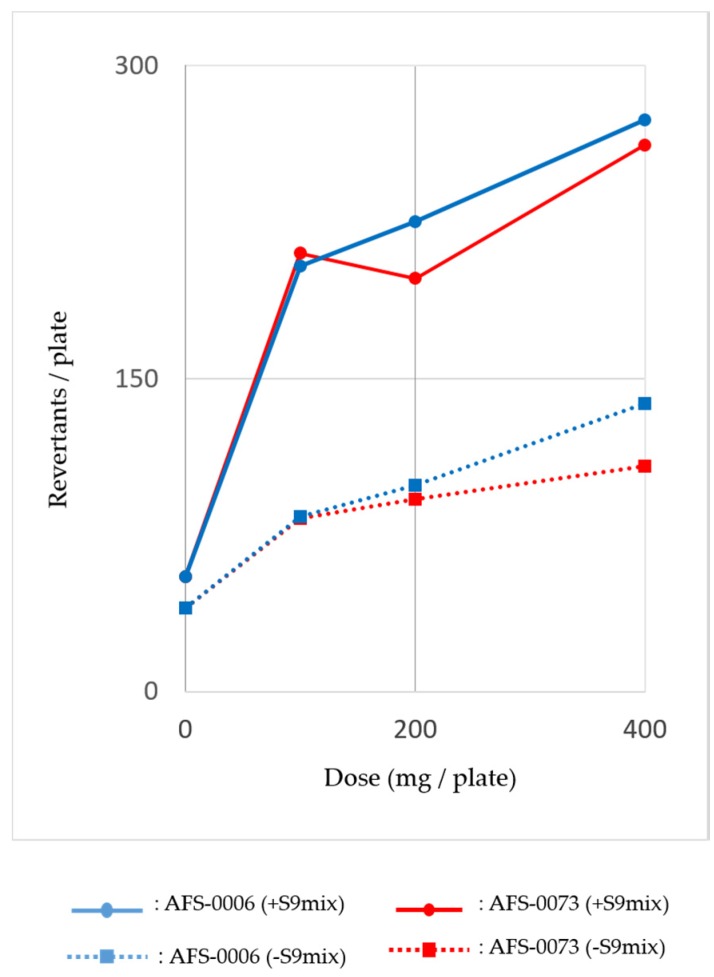
Mutagenicity of the methanol extract from *Penicillium brocae* for *Salmonella typhimurium* YG7108 with (●) and without (■) rat liver S9 mix.

The assay dose was calculated from the extract amounts per 4 g of rice grains and prepared with serial dilutions. Assays were performed with and without S9 mix using the tester strains of *Salmonella typhimurium* and *Escherichia coli*. Results showed that the methanol extracts of both strains were positive against the TA100 and YG7108 strains of *S*. *typhimurium*, as base pair substitution mutations occurred primarily at one of the G-C pairs. The mutagenic activity of YG7108 was higher than that of TA100. Figure 4 shows the dose-response curve of mutagenicity of the methanol extracts from the *P. brocae* AFS-0006 and AFS-0073 strains for *S. typhimurium* YG7108, both with and without rat liver S9 mix. The mutagenicity increased dose dependently, especially when treated with the S9 mix. These data indicated the presence of alkylating agents in the *P. brocae* metabolites.

### 2.4. Mycotoxin Analysis

**Figure 5:**
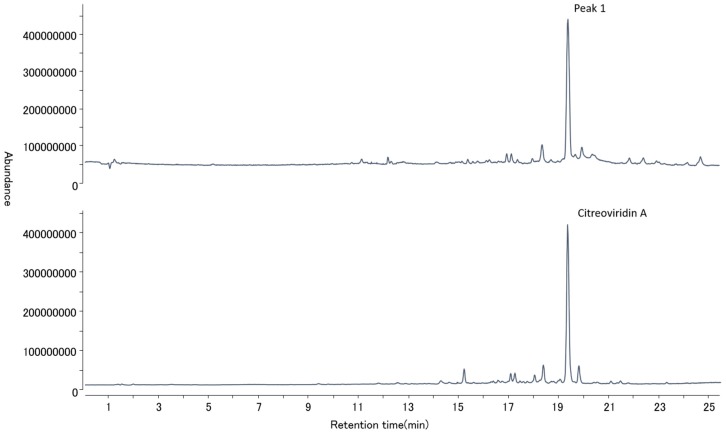
Total ion chromatograms of the *Penicillium citreonigrum* extract (upper) and citreoviridin A standard (lower) by LC/MS/MS.

**Figure 6:**
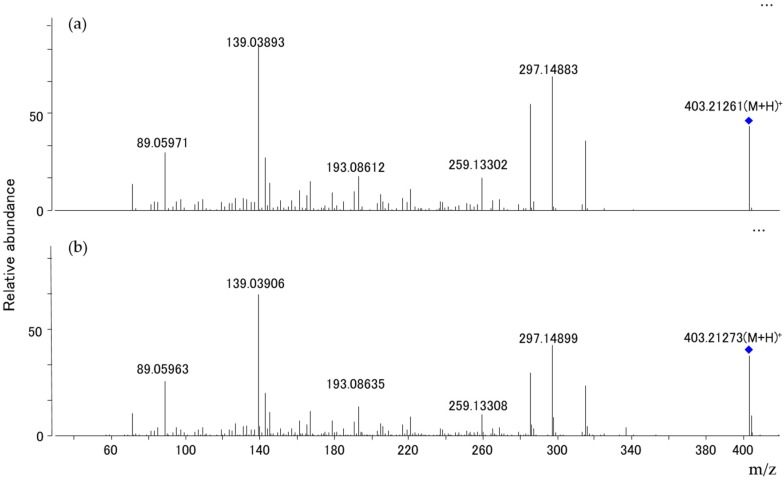
Accurate product ion spectra of peak 1 in Figure 5 and citreoviridin A standard. (**a**) Peak 1, (**b**) Citreoviridin A standard.

For the *P. citreonigrum* isolate obtained, an LC-QTOF analysis revealed that the isolate produced high amounts of CTV, when cultured on a yeast extract sucrose (YES) liquid cultural medium. Figure 5 and Figure 6 show the total ion chromatogram of the fungal extract, the accurate mass spectra of the main peak of the fungal extract, and citreoviridin A standard solution, respectively. The relative mass error of the protonated molecular ion at main peak was 2.71 ppm.

**Figure 7:**
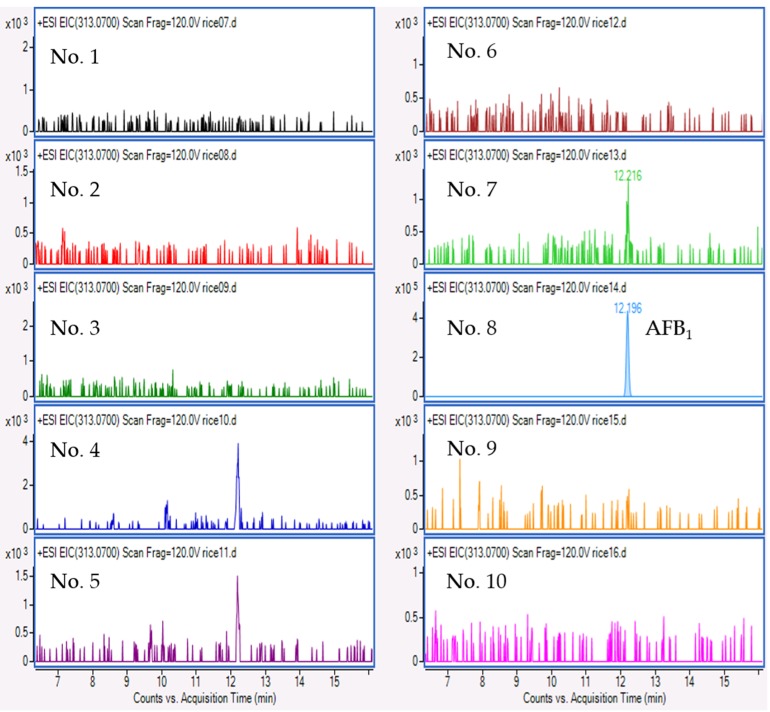
Extracted ion chromatograms of 10 rice samples for AFB_1_ detection.

Multiple mycotoxins survey showed that CTV was negative for any samples of Thai rice grains, although CTV-producing fungus was found. Among the 10 rice samples tested, AFB_1_ was detected at a concentration of 5.9 µg/kg in sample No. 8 obtained from the Pathum Thani region. Figure 7 shows extracted ion chromatograms of the rice samples for AFB_1_ detection. The other mycotoxins examined were not detected in any samples (data not shown).

## 3. Discussion

An incident involving rice with yellow coloration occurred in 1937 in Japan and was found to have been caused by *P. citreonigrum* [12]. The fungus produced a toxic metabolite, CTV, which is responsible for acute cardiac beriberi [17]. As beriberi disease did not occur in Japan after World War II, this yellowed rice toxic syndrome was largely forgotten. However, in 2006 to 2008, an outbreak occurred in Brazil due to the consumption of CTV-contaminated rice [18]. This incident reminded us of past issues associated with yellowed rice in Japan.

In this study, we obtained the target fungus *P. citreonigrum* from the surface of rice grains in Thai commodities. In addition, we also isolated *P. brocae* from the same samples, which was unexpected, as *P. brocae* is regarded as an endophytic fungus. These two *Penicillium* species showed slow growth on culture media.

In general, fungal isolation in rice grains is conducted with surface disinfection using chlorine, as several kinds of air-borne fungi may be present on the surface of rice grains. These contaminants can interfere with the surveillance of mycoflora on rice grains. However, we compared two methods in the present study, surface disinfection and direct plating without disinfection in order to examine the surface mycobiota on rice grains. We found toxigenic *P. citreonigrum* and endophytic *P. brocae*, along with other *Penicillium* species, only in the untreated group (Figure 1). As these two *Penicillium* species are not common air-borne fungi, our untreated approach seems to have been useful for the isolation of uncommon *Penicillium* species, possibly due to their susceptibility to the disinfecting agents compared with the common field or storage fungi of *Aspergillus*, *Bipolaris*, *Colletotrichum*, *Curvularia*, *Mucor*, *Phoma*, and *Rhizopus* [19]. This may explain why previous studies on mycoflora in rice failed to find any *P. citreonigrum* or *P. brocae*, except in the case of the *P. citreonigrum* responsible for food poisoning in Brazil [18].

An isolate of *P. citreonigrum* identified morphologically was obtained from a rice sample in the Surin region, which is in the northeast of Thailand, close to Cambodia. It produced CTV on YES medium. Kim et al. [20] reported that *P. toxicarium* was an endophytic fungus, because it was isolated from healthy needles of *Pinus rigida*, along with *Penicillium fellutanum*. *P. citreonigrum* and *P. toxicarium* are very close in the phylogenetic tree (Figure 2), but the taxon remains unclear. *P. brocae* was isolated firstly from the coffee berry borer *Hypothenemus hampei* [16], but it is known to be an endophytic fungus, generally derived from the coffee plant [21], or the *Zyzzya* sp. sponge [22], or the marine mangrove plant *Avicennia marina* [23]. Two research reports have shown that insects carried experimentally toxigenic *Aspergillus* and *Penicillium* species on stored maize kernels [24] and wheat grains [25]. Therefore, although *P. citreonigrum* and *P. brocae* were both isolated from rice grains in this study, they may have been introduced to the rice by insects, such as grain weevils, which tend to favor rice. Further research will be needed to confirm our speculation.

In the mutagenicity test, two *P. brocae* strains were tested to determine whether or not they could produce mutagens. The methanol extracts obtained from moldy rice grains were positive for base-pair substitution mutation and DNA alkylation using *S. typhimurium* strains TA100 and YG7108, respectively. Interestingly, these strains suggested the mutation of GC sites. As the mutagenic activity in the YG7108 strain was high, the data revealed that the metabolites of *P. brocae* AFS-0006 and AFS-0073 strains induced the alkylation of the GC sites (Figure 4). Further studies are underway to clarify the mutagenic agents produced by *P. brocae*.

Our fungal survey showed that toxigenic monoverticillate *Penicillium* species were present in commercially available Thai rice grains. However, CTV produced by *P. citreonigrum* was negative in any rice samples tested. AFB_1_ alone was detected in the No. 8 rice sample, although toxigenic *Aspergillus* species were not found in the sample (Figure 1). The culture plate of the No. 8 sample was fully covered with *Mucor* or *Rhizopus*. Anti-fungal chemicals, dichloran and rose bengal were inactive in depressing the growth of these fungi in the sample. Aflatoxin contamination in Thai commodities has been reported by several studies [2,8]. In this study, we also detected AFB_1_ in a sample of Luem phua rice in the Pathum Thani region, close to Bangkok. Although our survey was admittedly limited, the level of AFB_1_ contamination was higher than that in previous reports. Levels of contamination with AFB_1_ may depend on rice types.

## 4. Conclusions

Commercially available Thai rice samples were contaminated with several fungal species. Most of the species observed were not toxic, but a few toxigenic fungi were present, such as *P. citreonigrum* and *P. brocae*. Of note, these toxigenic *Penicillium* species possessed good ability to produce CTV or a mutagen. Thus, these species could be a potential threat to human health if environmental conditions are suitable for their growth.

## 5. Materials and Methods

### 5.1. Sample and Fungal Strain

Ten samples of rice were purchased from nine markets in Thailand. The types of rice and market regions were as follows: Jasmine rice (No. 1) in Bangkok, Dong Ruk rice (No. 2) in Surin, Pratum rice (No. 3) in Ayuddhaya, Riceberry gaba (No. 4) in Nakorn Pathom, Jasmine rice (No. 5) and brown jasmine rice (No. 10) in Chiang Rai, Red jasmine rice (No. 6) in Chaiyaphum, Organic brown rice (No. 7) in Chaing Mai, Luem phua rice (No. 8) in Pathum Thani, and Riceberry (No. 9) in Petchabune. These rice samples were chosen randomly, and packed into sealed plastic bags. The bags were kept closed until use. The samples sizes of the rice grains were ca. 1 kg each. 

As a reference strain, the *P. citreo-viride* IMI 92228 strain was obtained from the culture collection of CABI in the United Kingdom.

### 5.2. Fungal Isolation and Morphological Identification

One hundred rice grains from all 10 samples were used, and treated in two different manners: 50 grains were exposed to 70% ethanol solution for 30 s and then washed twice with sterile distilled water, and the other 50 grains were not treated in any manner. Following this treatment, five rice grains per plate were placed onto dichloran rose bengal chloramphenicol agar (DRBC) or dichloran 18% glycerol agar (DG18) medium and cultured at 25 °C for 5 days. After incubation, the genera of the developed fungi were simply classified by a macroscopic observation [26]. Among them, *Penicillium* species, showing monoverticillate and yellow pigmentation, were transferred to CYA and MEA media (BD, Sparks, MD, USA) and cultured at 25 °C for 7 days. According to the taxonomic criterion of the *Penicillium* species [26], the isolates were morphologically identified based on colony textures and microscopic observation.

### 5.3. Molecular Phylogenetic Analysis

A molecular phylogenetic analysis was carried out as follows. Fungi were pre-cultured on potato dextrose (PD) broth medium (Difco Laboratories, Franklin Lakes, NJ, USA) at 25 °C for 3–4 days. The genomic DNA was extracted from fungal mycelia by the sodium dodecyl sulfate method [27]. The *β-tub* regions were chosen for the phylogenic analysis. PCR amplification was done using Bt2a and Bt2b primers [28]. Sequencing was performed using a BigDye Terminator v3.1 Cycle Sequencing Kit and ABI3130 DNA Analyzer (Applied Biosystems, Foster City, CA, USA) according to the manufacturer’s instructions. Forty-five sequences of *β-tub* regions of the various *Penicillium* species and *Aspergillus niger* were downloaded from GenBank and used as reference data for the phylogenetic analysis. The species were selected with regard to various *Penicillium* clades reported by Visagie et al. [29] and *A. niger* was used as an outer species. The sequences were automatically aligned and the alignments were manually adjusted using MEGA6 [30]. A phylogenetic tree was constructed with the neighbor joining method by the maximum composite likelihood model using MEGA6.

### 5.4. Mutagenicity Assay

The mutagenicity assay was performed using *S. typhimurium* strains TA98, TA100, TA1537, and YG7108, and *E. coli* strain WP2/uvrA/pKM101 with and without rat liver S9 mix. These strains were chosen for detecting frameshift mutations (TA98 and TA1537) and base-pair substitution mutations (TA100, YG7108 and WP2), and YG7108 was particularly sensitive for DNA alkylation. The assay was tested for methanol extracts obtained from moldy rice grains, which were individually inoculated with tester isolates and cultured at 25 °C for 14 days. In accordance with the official guide of the mutagenicity test using microorganisms [31], which was revised by the Ministry of Health, Labor and Welfare of Japan in 1991, along with the method of Yamada et al. [32] for the *S*. *typhimurium* YG7108 strain, the assay was performed at five doses with two plates per dose for each sample. In addition, mutagenic standard chemicals were used as positive references against tester strains. The experiments were repeated twice, and the mutagenic activity was calculated from the slope of the linear portion of the dose-response curve using the statistical model of least squares linear regression.

### 5.5. Mycotoxin Analysis

For the CTV analysis, the tester isolate was cultured on YES liquid medium at 25 °C for 10 days. The CTV extract was then obtained in accordance with the method described by da Rocha et al. [13]. In target MS/MS acquisition for CTV, a data rate of three mass spectra per sec in MS and MS/MS mode was used. Target compounds were detected and reported from accurate-mass data using Agilent Mass Hunter Qualitative and The Agilent Mycotoxins and Related Metabolites Personal Compound Database and Library (PCDL) software programs. The PCDL database contains 455 target compound names, molecular formulae, CAS numbers, structure and 297 MSMS spectra. The Qualitative Analysis software program tentatively identified target compounds using a “Find by Formula” algorithm with the following criteria: relative mass error, 5 ppm; isotope abundance and spacing score, greater than 90 out of 100. The reagent of citreoviridin A standard used was obtained from Fermentek Ltd. (Jerusalem, Israel).

Fifty grams of rice grains from each sample were powdered using a blender machine, and a 7.5 g portion of the powder sample was weighed and transferred to a 50-mL centrifuge tube. Mycotoxins were extracted using the QuEChERS extraction kits (Agilent Technologies, Inc., Tokyo, Japan) with slightly modified citrate buffer. For clean-up, a dispersive solid-phase extraction (Agilent Technologies, Inc.) was used. In accordance with the manufacturer’s protocol, the purified extracts obtained were analyzed by liquid chromatography coupled with quadrupole time-of-flight mass spectrometry (LC-QTOFMS) equipped with a dual-spray Jet Stream electrospray ionization source. The LC system was an Agilent 1290 Infinity II LC system (Agilent Technologies, Inc.) consisting of binary pumps, degasser, column compartment and autosampler with a reverse-phase column of ZORBAX Eclipse plus C_18_ (100 × 2.1 mm, 1.8 μm) and operated with a gradient mobile phase containing 0.1% formic acid + 10 mM ammonium acetate (A) and acetonitrile, or methanol (B). The initial combination was 90% A and 10% B, changing linearly to 0% A and 100% B over 30 min. The flow rate was 0.2 mL/min. 

The quadrupole time-of-flight mass spectrometry system was the Agilent 6550 iFunnel Quadrupole Time-of-Flight LC/MS (Agilent Technologies, Inc.) operated in positive ion mode with a mass ranging from 100 to 1000 Da and a data rate of 1.5 mass spectra per sec. The instrument acquired data using the following parameters: drying gas temperature, 200 °C; drying gas flow, 20 L/min; nebulizer, 50 psi; sheath gas temperature, 400 °C; sheath gas flow, 12 L/min; VCap. 4000 V; nozzle, 0 V; fragmentor, 360 V; skimmer, 65 V; and octapole RF peak, 750. A constant flow of Agilent TOF reference solution containing purine (*m*/*z* = 121.05087) and HP-921 (*m*/*z* = 922.00979) through the reference nebulizer allowed the system to continuously correct for any mass drift.

Twenty-six mycotoxins; AFB_1_, AFB_2_, AFG_1_, AFG_2_, AFM_1_, citrinin, CTV, cyclochlorotine, cyclopiazonic acid, deoxynivalenol (DON), DON-glucoside, 3-acetylDON, 15-acetylDON, diacetoxyscirpenol, fumonisin B_1_, fumonisin B_2_, luteoskyrin, nivalenol (NIV), NIV-glucoside, 4-acetylNIV, ochratoxin, patulin, sterigmatocystin, T-2 toxin, HT-2 toxin, and zearalenone, were examined by LC-QTOFMS.
